# Supplementary material for: Antimicrobial and Immunomodulatory Effects of Selected Chemokine and Antimicrobial Peptide on Cytokine Profile during Salmonella Typhimurium Infection in Mouse
Source: Antibiotics (Basel). 2022 Apr 30;11(5):607. doi: 10.3390/antibiotics11050607 (PMC9137564; doi:10.3390/antibiotics11050607)
Supplement: Supplementary file 1 [file antibiotics-11-00607-s001.zip › antibiotics-1631687-supplementary.pdf]

antibiotics

MDPI

Supplementary material

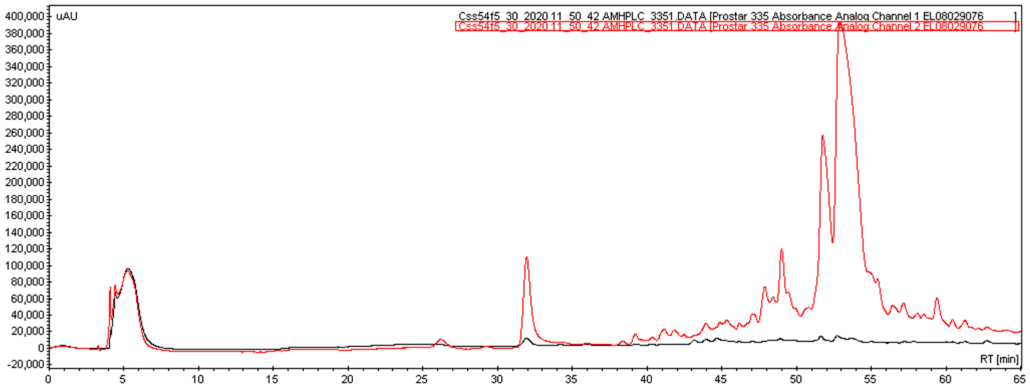

Figure S1. Purification of Css54. A C18 column (4.6 x 250 mm) was used with a 0-60% B gradient in 65 min, flow 1 mL / min. Solvent A: H2O + 0.1% TFA; Solvent B: Acetonitrile + 0.1% TFA. Css54 retention time: 52 min (47% B).

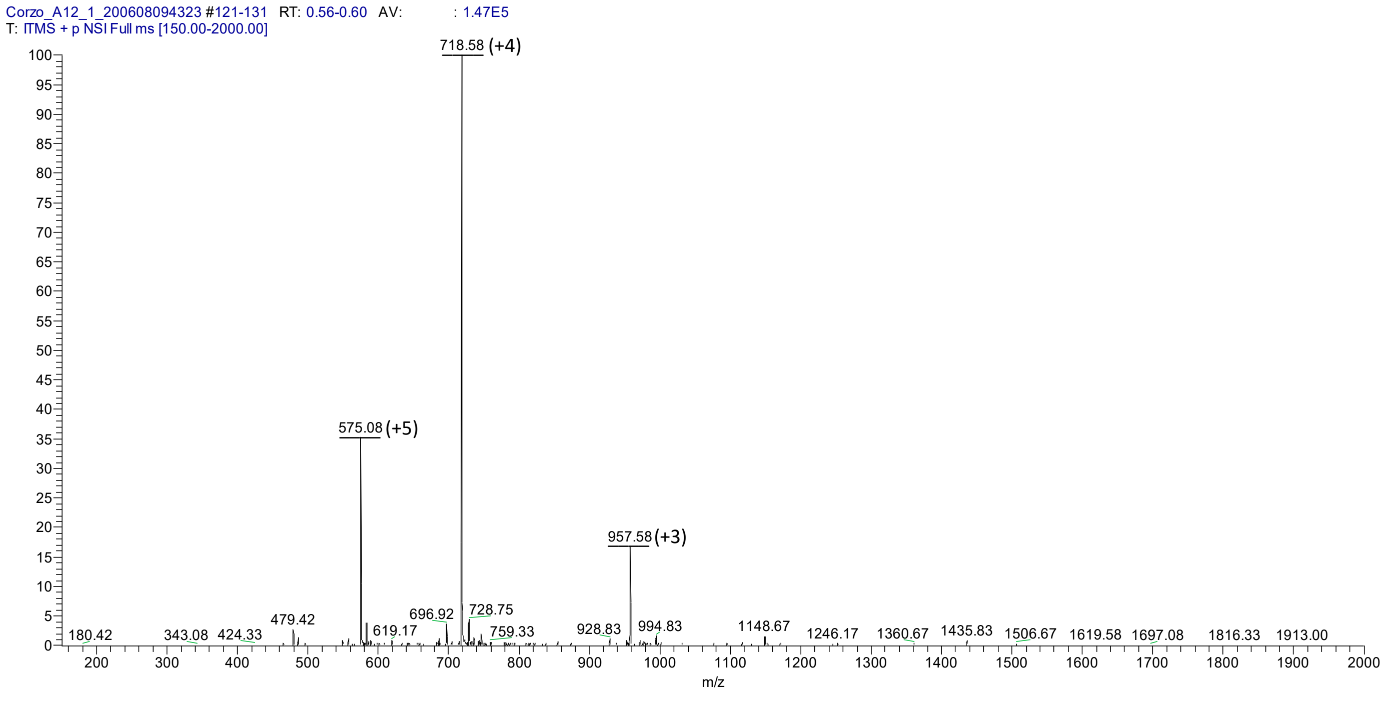

Figure S2. Mass spectrometry analysis of Css54.

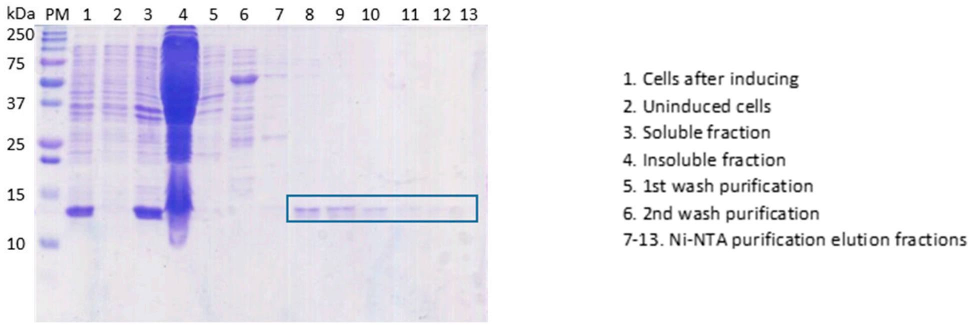

Figure S3. 15% SDS-PAGE gel showing MCP-1 expression and purification.

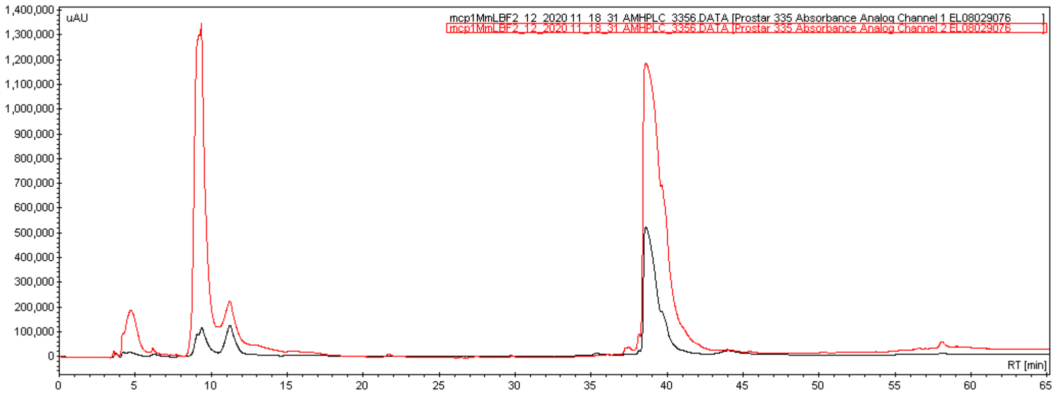

Figure S4. Purification of MCP-1 by RP-HPLC. Column C4 (4.6 x 250 mm). Gradient 0-60% B in 60 min, flow 1 mL / min. Solvent A: H2O + 0.1% TFA; Solvent B: Acetonitrile + 0.1% TFA. MCP-1 retention time: 37.8 min (32.8% B). The traces represent the absorbance at 230 (red) and 280 (black) nm, respectively.

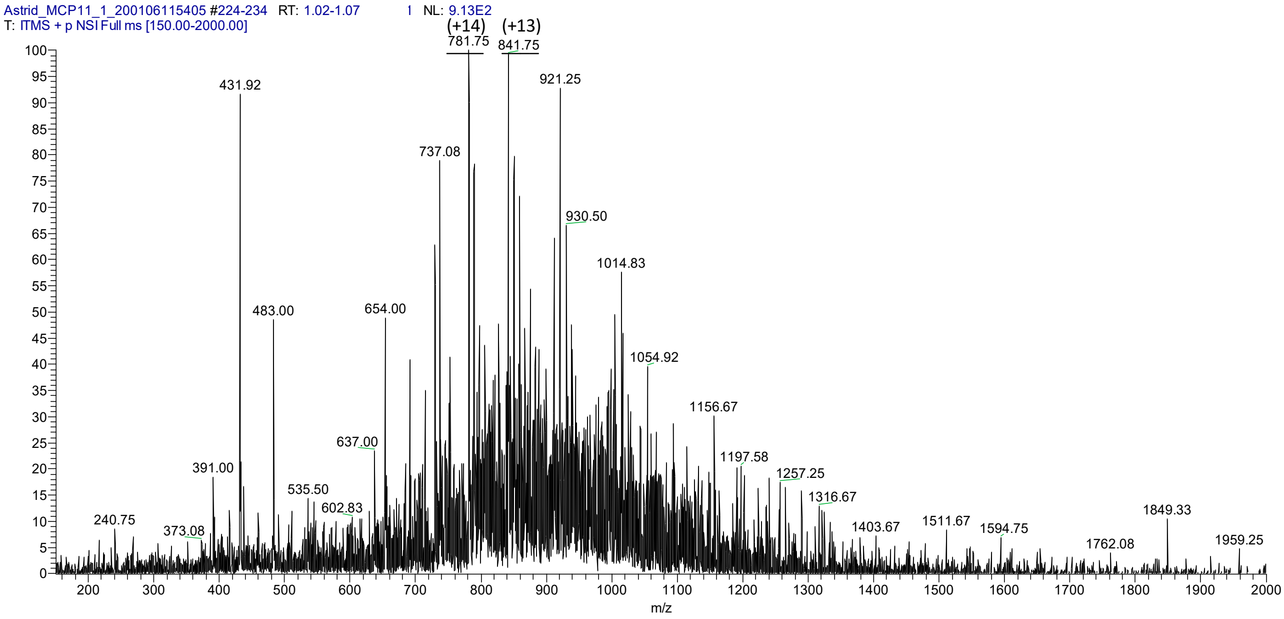

Figure S5. Mass spectrometry analysis of MCP-1.
